# Dr. Walker, on Our Perceptions of Time

**Published:** 1808-08

**Authors:** 


### *To the Editors of the Medical and Physical Journal.*

Gentlemen,

In taking up the pen, I anticipate that the two or three ideas I shall now have to offer to you, bearing more or less directly upon animal physiology, though not strictly medical, may be received into your Journal. Perhaps you will think *nihil humani alienum* from that most curious of all subjects of contemplation.

Sitting this morning by a clock, and happening to notice, particularly, the beating of the seconds, I was forcibly struck with the recollection of being once, similarly, engaged above forty years ago, when a child, and left alone, on a visit in the country. At that time, the oscillations of the pendulum seemed to me exceedingly slow; now, they seem extremely quick. About that time the tune of '*Hearts of Oak*' was indelibly impressed upon my sensorium, from its being continually played in the processions, at a long contested election. We used, little joyous school-boys, with quickened step, to keep up with the processions. It then seemed to me a very slow going tune; during one note, we must have had to paddle forward two or three steps, while the electioneers, in their ordinary step, perhaps, marched in time. I was lately at a public dinner, when a band struck up this tune, and I was astonished at its rapidity; but I suspected at the time, that the change had been brought about, during the course of forty annual suns, in my own feelings; that time itself, the shortest portion of it, seemed shorter to me now than it did in childhood.

childhood. The ordinary step of men in walking would have beat time to the tune, as I suppose it did when I was a little boy. My observations on the clock this morning, help to confirm me in the opinion. I believe a second of time may, naturally, appear to a child as long a portion of it as one-thirtieth, or even one-twentieth, of a minute may seem to him, who has lived, in this world, nearly half a century.

Do not the observations of all the world agree with these statements of the apparent change, the seemingly continually-accelerating rapidity of time, during active human life? Leave of absence from duty for any number of months to a veteran in service, is not to be compared with a holiday of so many weeks to a school-boy, or indeed of so many days, if he be but young enough. I remember, perfectly, on the eve of a Christmas vacation, being astonished in looking forward at it. It seemed to me a duration of almost interminable length. Enraptured, I recoiled, on myself, from the attempt to comprehend the immense field of gay pleasure which lay before me.

Do we not, generally, find our years to become shorter and shorter to us, the longer we live? On our first rising into life, looking about us, and making our observations, every thing is new; the feeling of our own existence we have scarcely yet got inured to. Life itself seems a novelty to us. It has not yet become insipid through our merely having been long accustomed to it, which seems so often to happen to those who, without objects of pursuit, are consumed with *ennui*. But the thread of human life, while it seems to be thus, generally, spun out to us individually with a continually accelerating rapidity, does evidently put on some considerable varieties, accordingly as our condition in life may be affected or diversified. The domestic character, when he first enters upon travelling, finds his days and weeks seemingly lengthened out to a duration equal to what they ever had been at the time of his childhood, when every object was new to him; while the traveller, on the other hand, detained by a contrary wind, feels a tedium in his condition that often makes the time hang heavily on his hand till he again gets under weigh; and both Plaintiff and Defendant, during a weary law-suit, sometimes find their days and nights of greater length than they ever experienced them before.

I know not whether these conditions of humanity, these modifications of our perception of time, more particularly that apparent shortening of it as we advance in life, be increasedly creasedly continued till old age. I have observed in people, advancing far in years, that they have had a more lively recollection of the scenes of their early life than of those of later times; perhaps, at this time, their most highly cultivated faculties beginning to be impaired, their days and weeks do not seem to hurry over with that rapidity which they did in more active life. With all their former associations, perhaps, their days have also obtained their former duration; and while they have to give so much of them to sleep, their vigils may appear lengthened to them. If this be the case, the condition of age, when not affected with disease or pain, may be more desirable than we might otherwise suppose. Even when dotage, at length, arrives, it brings a condition which, perhaps, might quite as fitly excite a feeling of envy as of pity. Such subjects I have observed to gradually, or regularly, trace back the scenes of their former life.—Present affairs escaped their notice; they thought and spoke only of those which had occupied their attention before the failing of their recollection. As they advanced further in years, they retrograded into active life, dwelt upon their pursuits at that time, and fancied themselves still engaged in them. Further advancement in years, brought the recollection of the scenes of their youth; they kept talking of their associates of those days, and seemed to enjoy themselves. A further continuance of life, brought them to a second childhood; all their former dispositions seemed to return to them; and they failed not to be, again,

——————— by Nature's kindly law,

Pleas'd with a rattle, tickled with a straw.

Even disease, sometimes, debilitates the mental powers, and prematurely, and only *pro tempore*, brings about these effects; of which the following little narrative, which I have heard related, both in this country and in Ireland, affords a very striking example.

A patient, much reduced under typhus, kept muttering in a way which his attendants could not understand. A Welch woman coming within hearing of him, told them that she understood every word he said; that it was all childish talk in her own native language. The patient recovered, and could only speak English. On enquiry, it appeared that when a child, his nurse, who was a Welch woman, had learned him to prattle to her in her native tongue; and in the reduced state of the system, which typhus had induced, his later acquirements had been suspended, and those of his childhood, only, had returned upon him. J. W.

*Salisbury Court, 9, vij, 1808.*

